# Supplementary material for: Retrotransposon activation contributes to neurodegeneration in a Drosophila TDP-43 model of ALS
Source: PLoS Genet. 2017 Mar 16;13(3):e1006635. doi: 10.1371/journal.pgen.1006635 (PMC5354250; doi:10.1371/journal.pgen.1006635)

Repo	KEGG ALS	Other	Row total
DE	9	1585	1594
Non-DE	13	6069	6082
Column total	22	7654	7676

Fisher's exact test
p-value < 0.0312

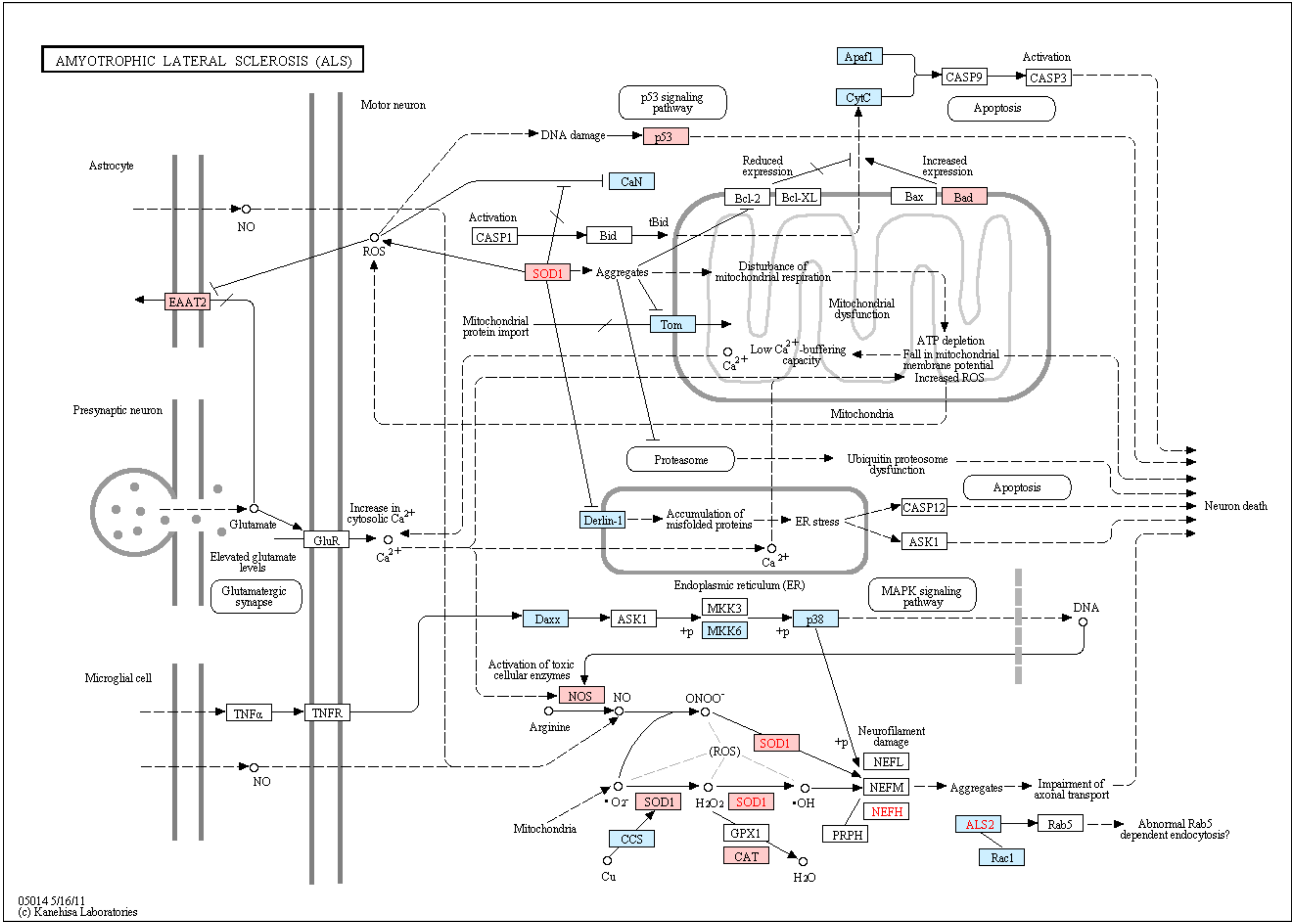

Supplement: S1 Fig — Among the KEGG gene set annotated as functionally related to ALS pathways. Although this is by no means a complete list of genes that have been implicated in ALS in the literature, it represents a set of gene pathways with known involvement in ALS. 20 of the ALS KEGG gene set contain clear Drosophila orthologs and a significant fraction of these (11/20) are identified as differentially expressed in our RNAseq from Repo>TDP-43 heads. (PDF) [file pgen.1006635.s001.pdf]
