# Supplementary figures and images for: Retrotransposon activation contributes to neurodegeneration in a Drosophila TDP-43 model of ALS
Source: PLoS Genet. 2017 Mar 16;13(3):e1006635. doi: 10.1371/journal.pgen.1006635 (PMC5354250; doi:10.1371/journal.pgen.1006635)

Figure S2A–S2B.

a.1 ELAV > hTDP-43

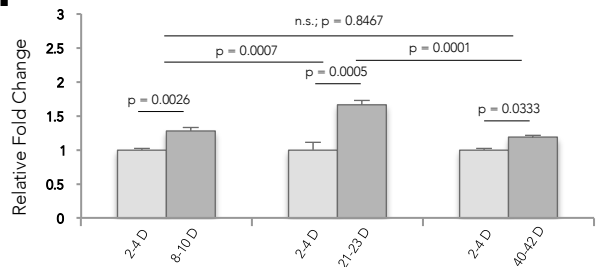

a.2 ELAV / +

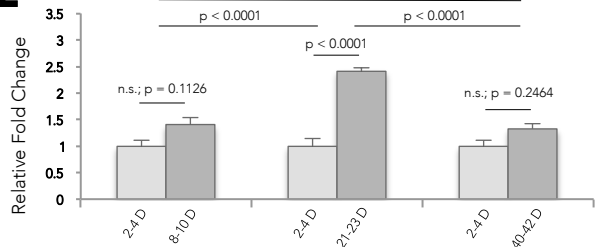

a.3 hTDP-43 / +

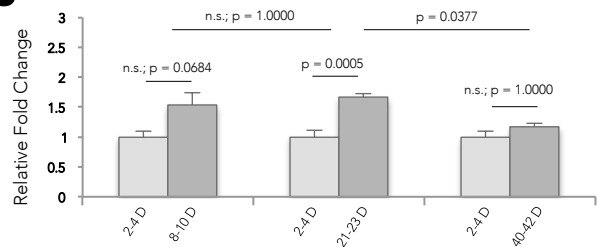

a.4

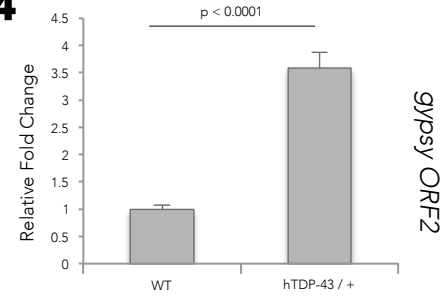

b

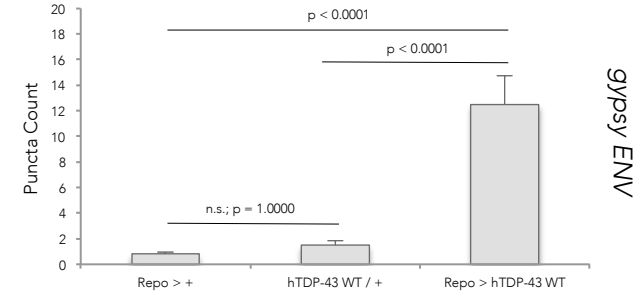

a.5

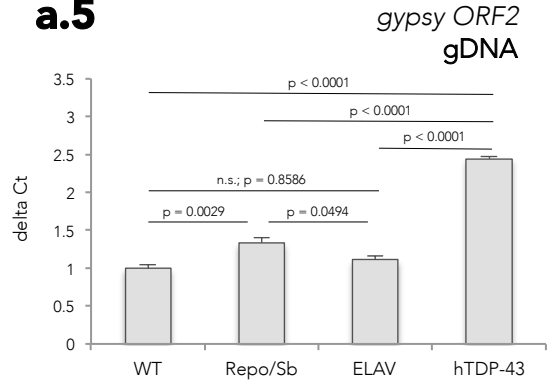

a.6

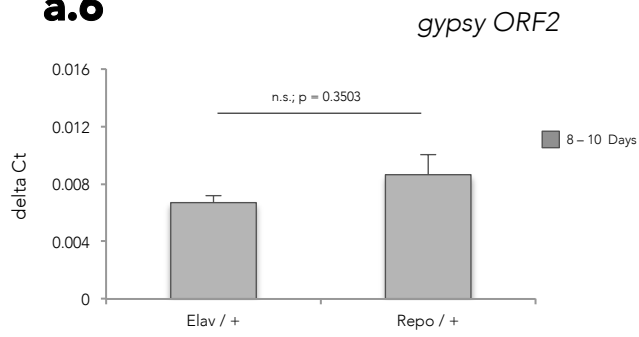

**Figure S2C.**

**C.**

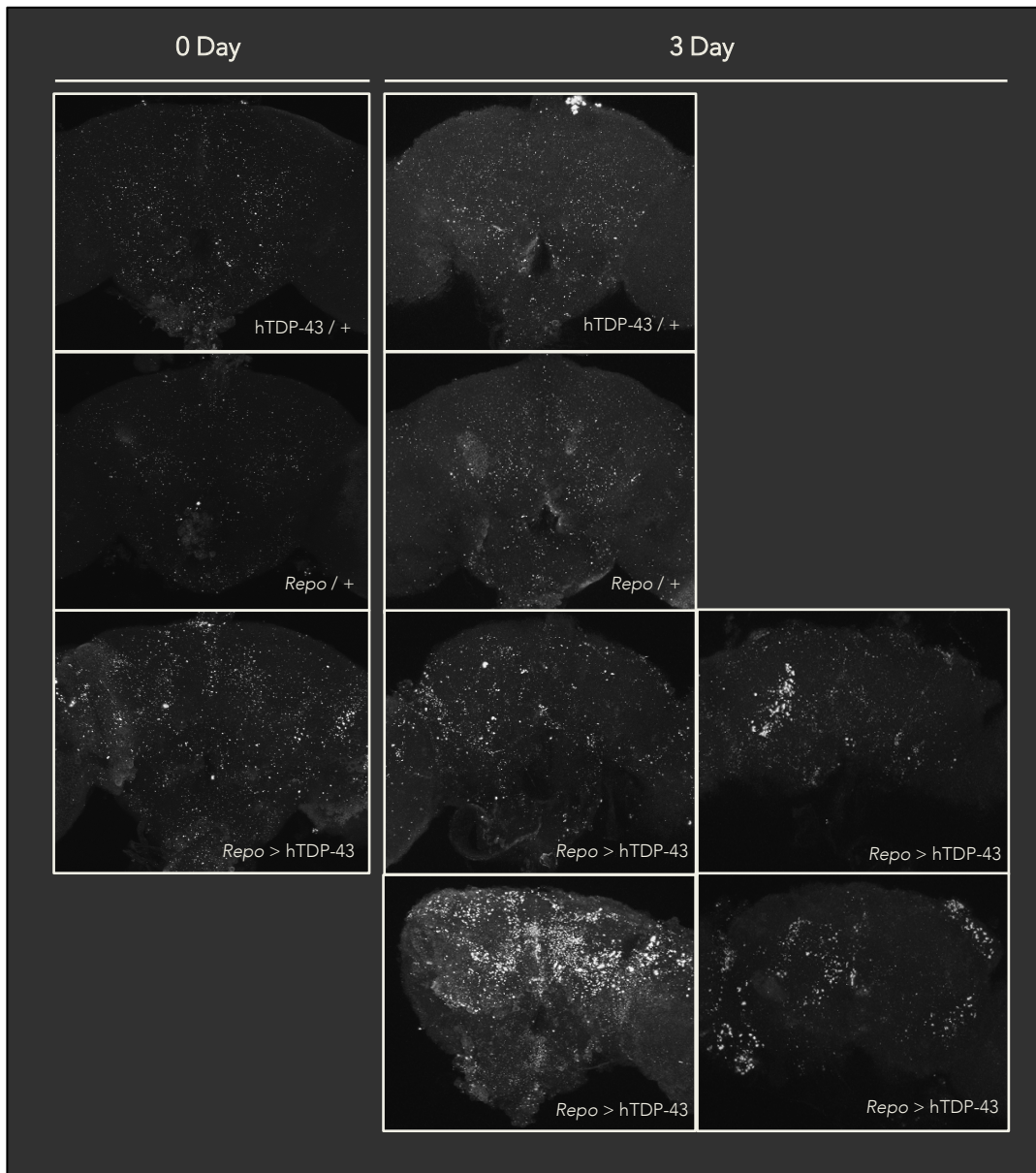

Supplement: S2 Fig — (A) Transcript levels of gypsy ORF3 (Env) as detected by qPCR on whole head tissue of flies expressing (A.1) neuronal hTDP-43 (ELAV > hTDP-43), or genetic controls: (A.2) ELAV / + and (A.3) hTDP-43 / +. gypsy ORF3 transcript levels display an increase by 21–23 days post-eclosion that drops back down by 40–42 days post-eclosion, regardless of genotype. In all cases transcript levels have been normalized to Actin, and the aged cohort (8–10 days; 21–23 days; 40–42 days) are represented as a fold change over an appropriate young (2–4 day) cohort that has been processed in parallel. Unpaired t-tests have been used to calculate p-values for each aged cohort with its matched young cohort, while p-values comparing aged cohorts within genotypes have been calculated using the Bonferroni method for multiple comparisons. For all three genotypes a one-way ANOVA shows a significant effect of age on gypsy ORF3 transcript levels between the aged cohorts (ELAV > hTDP-43, p < 0.0001; ELAV / +, p < 0.0001; hTDP-43 / +, p = 0.0346). N = 5 for all groups. (A.4). qPCR of whole head tissue reveals that the presence of the hTDP-43 transgene alone with no Gal4 driver results in elevation of gypsy ORF2 transcript levels. N = 6 for both groups. Quantitative genomic PCR (A.5) reveals that the wild type, Elav-Gal4 and Repo-Gal4 lines have comparable levels of gypsy DNA copy number, and (A.6) mRNA expression levels. The UAS-hTDP-43 parental line exhibits marginally higher levels of gypsy genomic DNA (A.5), although this cannot explain the difference between expression in ELAV > hTDP-43 vs Repo> hTDP-43. (B) Quantification of immunoreactive puncta averaged across the central 10 optical slices of brains of flies expressing hTDP-43 in glial cells (Repo > hTDP-43) and genetic controls (Repo / + and hTDP-43 / +) aged to 10 days and whole-mount immunostained using the gypsy ENV monoclonal antibody in a separate experiment from Fig 1C. A one-way ANOVA reveals that there is no difference in ENV immunore [file pgen.1006635.s002.pdf]

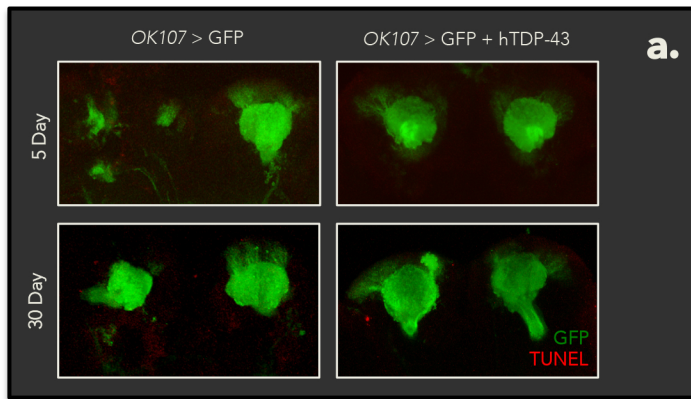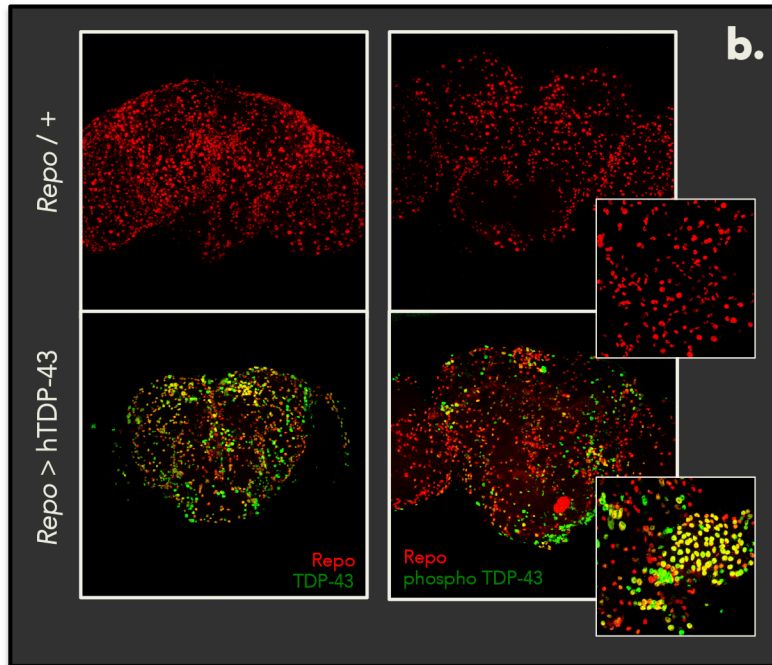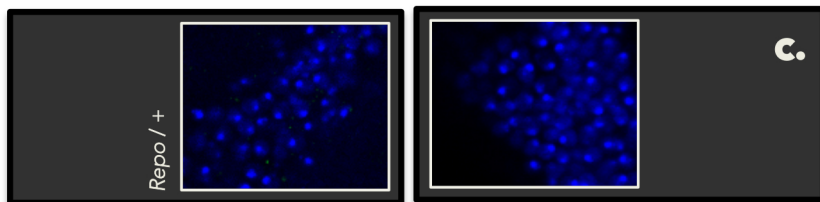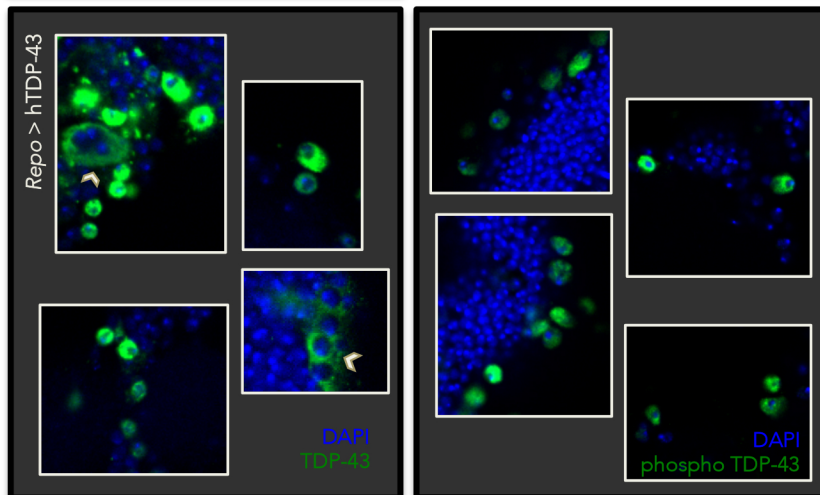

**d.** Repo > hTDP-43

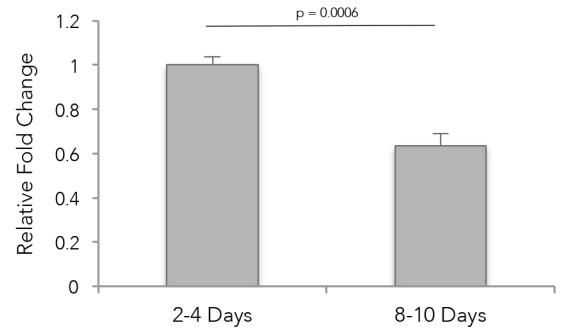

**e.** ELAV > hTDP-43

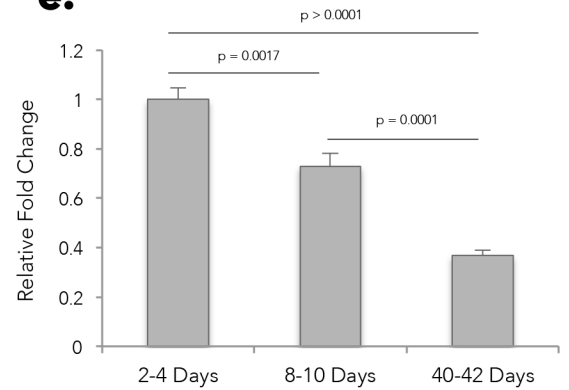

**f.**

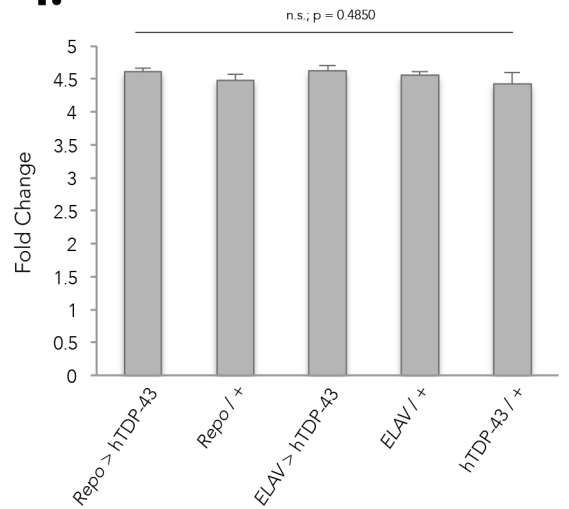

TARDBP

TBPH

Supplement: S3 Fig — (A) TUNEL staining reveals very little apoptotic activity when hTDP-43 is expressed in the mushroom body under OK107-Gal4, even when the animals are aged to 30 days post-eclosion. Mushroom bodies marked by co-expression of GFP, shown in green; TUNEL staining shown in red. OK107 > GFP, 5 Day, N = 3; OK107 > GFP + hTDP-43, 5 Day, N = 5; OK107 > GFP, 30 Day, N = 5; OK107 > GFP + hTDP-43, 30 Day, N = 4. (B) Full length human TDP-43 (green) can be detected by immunolabelling in the brains of flies expressing glial hTDP-43 under the Repo-Gal4 driver at 21–23 days post-eclosion, and co-localizes with Repo (red) immunoreactivity (left). Repo / + (N = 4); Repo > hTDP-43 (N = 4). Immunoreactivity for a disease-specific phosphorylated isoform of the protein (pSer409) can also be readily detected and co-localizes with Repo (right). Repo / + (N = 7); Repo > hTDP-43 (N = 4). A 63x blow-up is shown in the pop-out. (C) Both the full-length (left) and disease specific (right) isoforms of hTDP-43 (green) are mainly observed in the cytoplasm and vacate the nucleus (visualized by DAPI co-staining, shown in blue). Arrowheads indicate the hTDP-43-filled cytoplasm of a cortical glial cell wrapped around several neuronal nuclei in the neuropil of flies expressing glial hTDP-43. For full length hTDP-43 antibody, Repo / + (N = 6), Repo > hTDP-43 (N = 13); for pSer409 phosphorylated hTDP-43 antibody, Repo / + (N = 4), Repo > hTDP-43 (N = 9). (D) qPCR of whole head tissue demonstrates that transcript levels of hTDP-43 diminishes under Repo-Gal4 from 2–4 days to 8–10 days. Transcript levels normalized to Actin and displayed as fold change relative to 2–4 day old flies (means + SEM). N = 6 for all groups. (E) A similar effect of age on hTDP-43 expression is observed in neurons under ELAV-Gal4, and continues to drop off by 40–42 days post-eclosion. A one-way ANOVA shows a significant effect of age (p < 0.0001). N = 6 for all groups.(F) qPCR of whole head tissue demonstrates that expression of hTD [file pgen.1006635.s003.pdf]

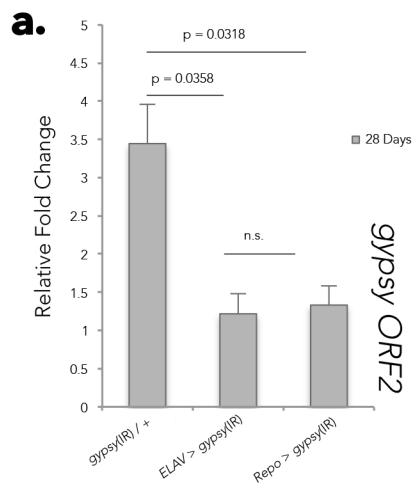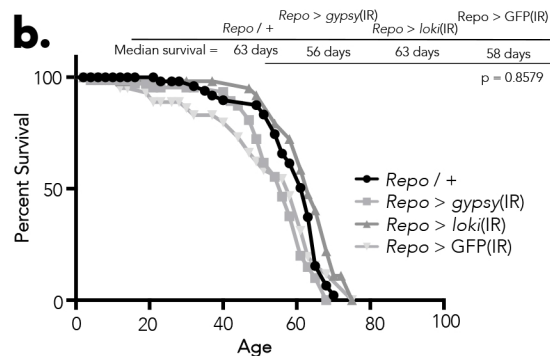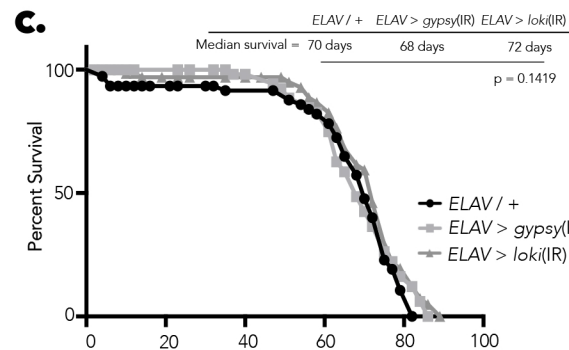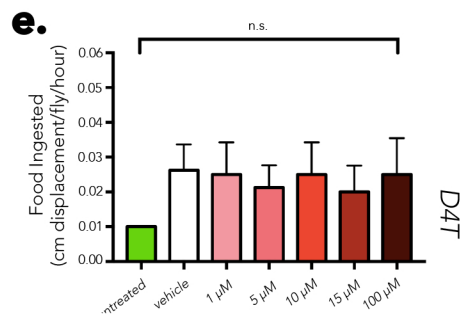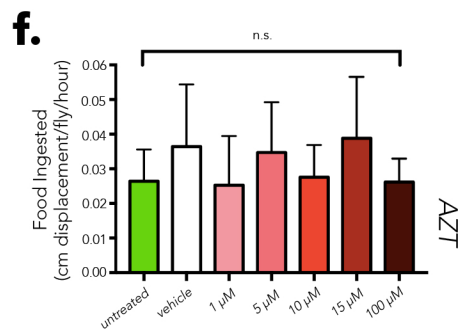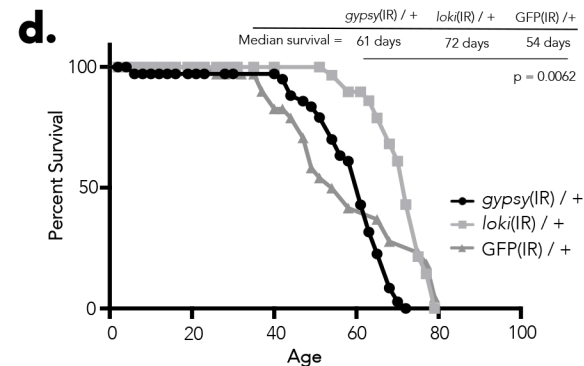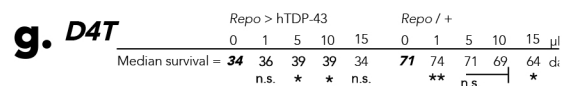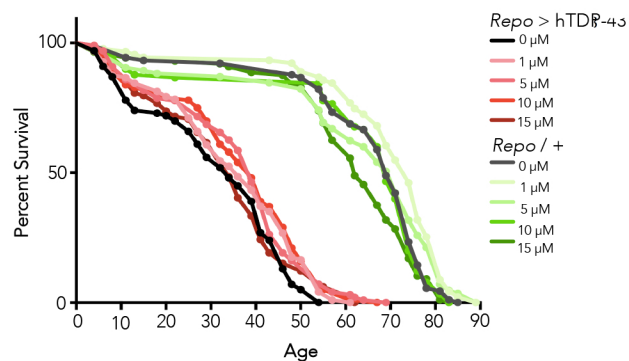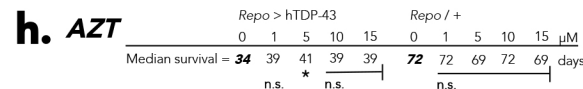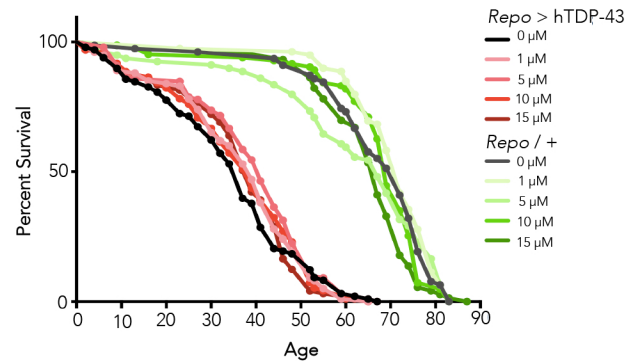

Supplement: S4 Fig — However, NRTI administration extends lifespan in flies expressing hTDP-43 in glia. (A) qPCR on head tissue demonstrates that expressing an IR directed against gypsy ORF2 (gypsy(IR)) in neurons (ELAV > gypsy(IR)) or glia (Repo > gypsy(IR)) effectively inhibits age-dependent elevation of gypsy transcript levels, and results in an ~2.5-fold reduction at 28 days post-eclosion. gypsy transcript levels from head tissue of young (2–4 day) and aged (28 day) flies was normalized to Actin and displayed as fold change in 28 day old flies of each genotype relative to each respective young cohort (displayed as means + SEM). A one-way ANOVA shows a significant effect of genotype (p = 0.0182). N = 2–3 biological replicates generated from heads of 5 mL of flies for each group. (B) Expression of gypsy(IR), loki(IR), and GFP(IR) individually in glial cells under the Repo-Gal4 driver does not significantly alter lifespan. (C) Expression of gypsy(IR) and loki(IR) individually in neurons under the ELAV-Gal4 driver does not significantly alter lifespan. (D) The presence of each of the IR constructs alone without any Gal4 driver (gypsy(IR) / +; loki(IR) / +; GFP(IR) / +) only moderately effects lifespan. (E) The capillary feeder (CAFE) assay demonstrates that neither the final concentration of vehicle (0.2% DMSO) alone, any of the final concentrations of D4T used in solid fly food in the lifespan analysis (Fig 4D; 1 μM, 5 μM, 10 μM, or 15 μM D4T in 0.2% DMSO), or an additional high concentration of D4T (100 μM in 0.2% DMSO) significantly altered displacement by consumption by wild type flies of a liquid media solution (5% sucrose / 5% autpolyzed yeast) (one-way ANOVA; p = 0.2137) over the course of 24 hours. Fly-less assay tubes containing vehicle solution alone were used to control for evaporation. Displayed as means + SEM; N = 6–8 for all D4T groups. (F) CAFE assay performed as in S5E Fig demonstrates that AZT does not significantly alter feeding behavior of wild type flies (p = 0.0595) [file pgen.1006635.s004.pdf]

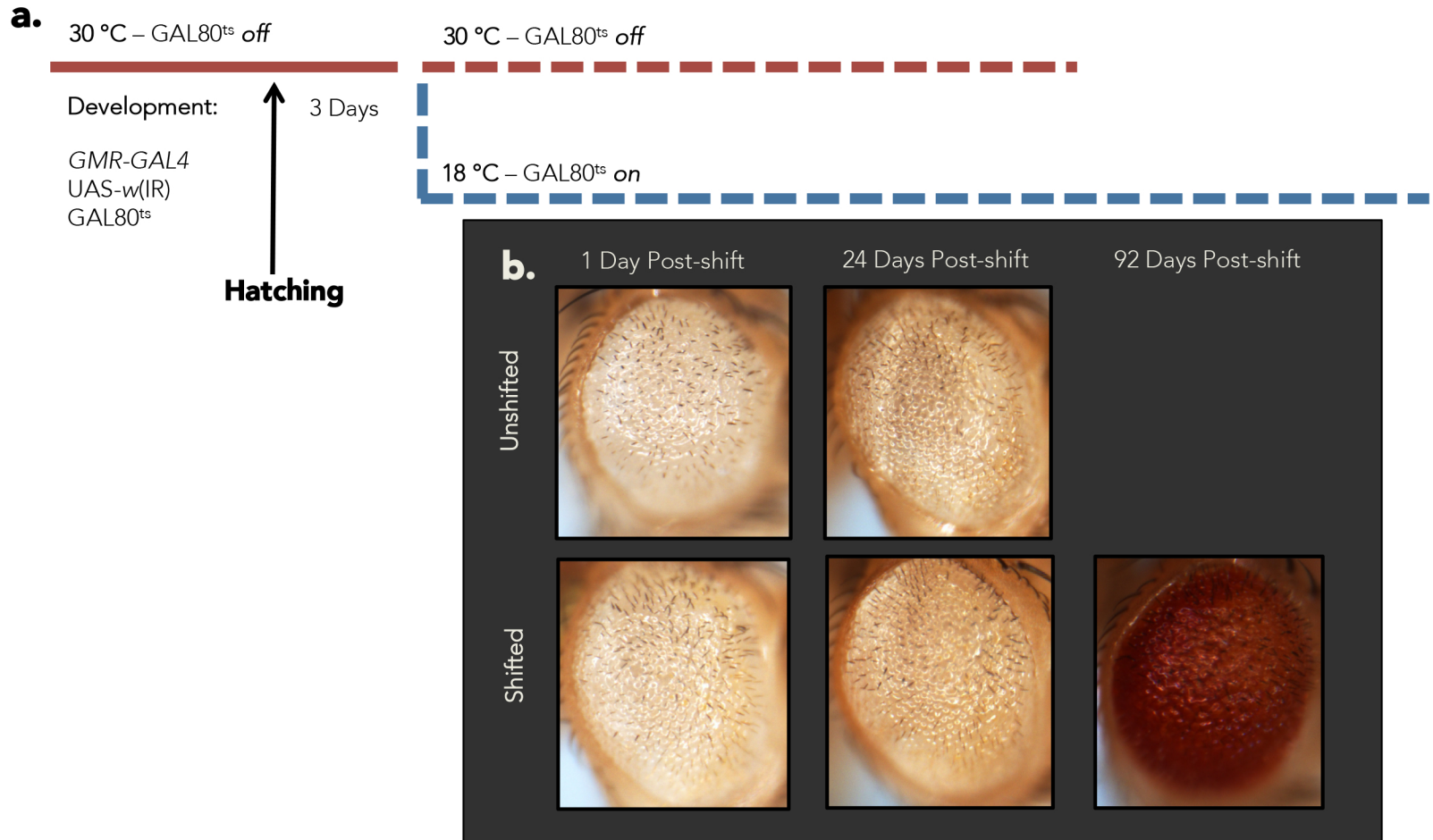

**c.1**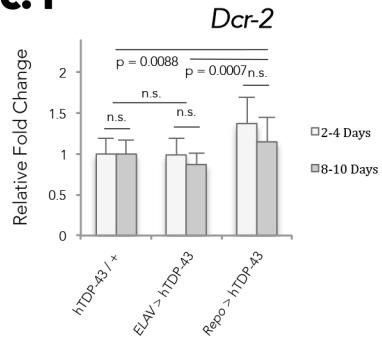**c.2**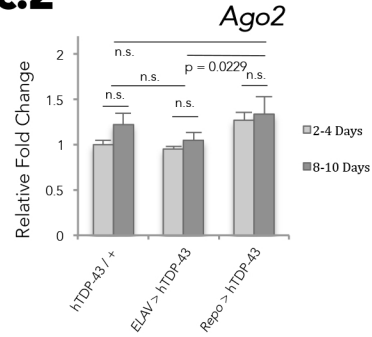**d.1****ELAV > hTDP-43**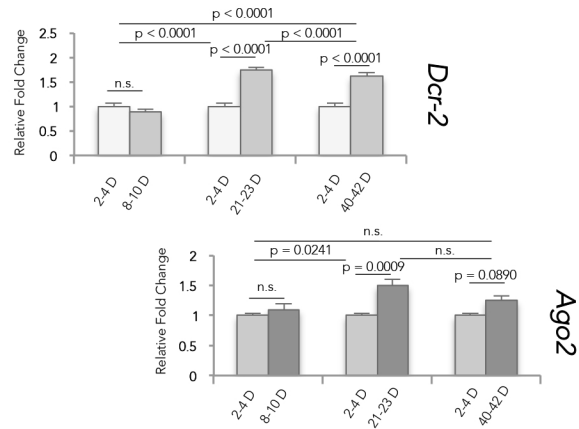**d.2****ELAV / +**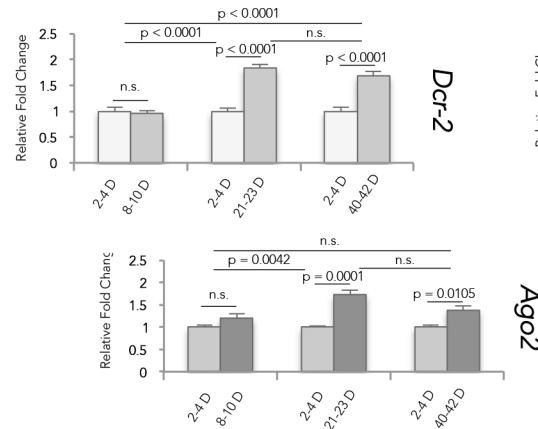**d.3****hTDP-43 / +**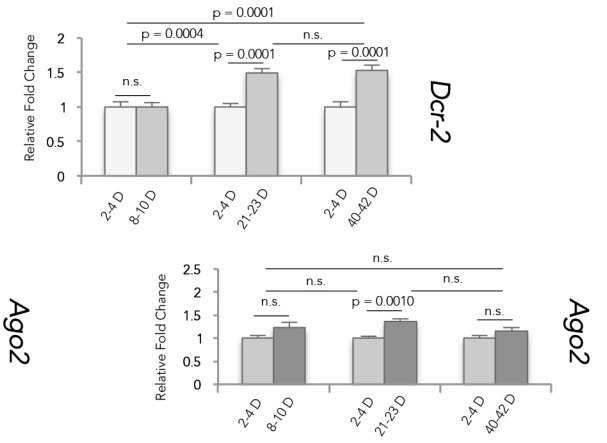

Supplement: S6 Fig — Loss of suppression of gypsy cannot be explained by hTDP-43- or age-dependent effects on siRNA effector molecules. (A) Schematic representation of the experimental design. (B) Representative images demonstrating that turning off w(IR) expression post-developmentally rescues red pigmentation of the Fly eye. N = 5 for all groups. (C) qPCR of whole head tissue demonstrates that reduced expression of (C.1) Dcr-2 and (C.2) Ago2 cannot account for loss of suppression of gypsy in flies expressing glial hTDP-43 (Repo > hTDP-43) at either 2–4 or 8–10 days post-eclosion. Transcript levels normalized to Actin and displayed as fold change relative to flies carrying the hTDP-43 transgene with no Gal4 driver (hTDP-43 / +) at 2–4 Days (means + SEM). For Dcr-2, a two-way ANOVA reveals an effect of age (p = 0.0006) but no effect of genotype (p = 0.1081); for Ago2, a two-way ANOVA also reveals an effect of age (p = 0.0258) but no effect of genotype (p = 0.1591). N = 8 for all groups. (D) qPCR of whole head tissue demonstrates that age-dependent changes in expression of Dcr-2 (top) and Ago2 (bottom) cannot account for age-dependent loss of suppression of gypsy in flies expressing neuronal hTDP-43 (ELAV > hTDP-43; D.1) or genetic controls: (D.2) ELAV / + and (D.3) hTDP-43 / +. All data analyzed as in (S1A.1-S1A.3); one-way ANOVA shows an effect of age across almost all groups (ELAV > hTDP-43, Dcr-2, p < 0.0001, Ago2, p = 0.0269; ELAV / +, Dcr-2, p < 0.0001, Ago2, p = 0.0051; hTDP-43 / +, Dcr-2, p < 0.0001, Ago2, p = 0.3967). N = 8 for all groups. (PDF) [file pgen.1006635.s006.pdf]

a

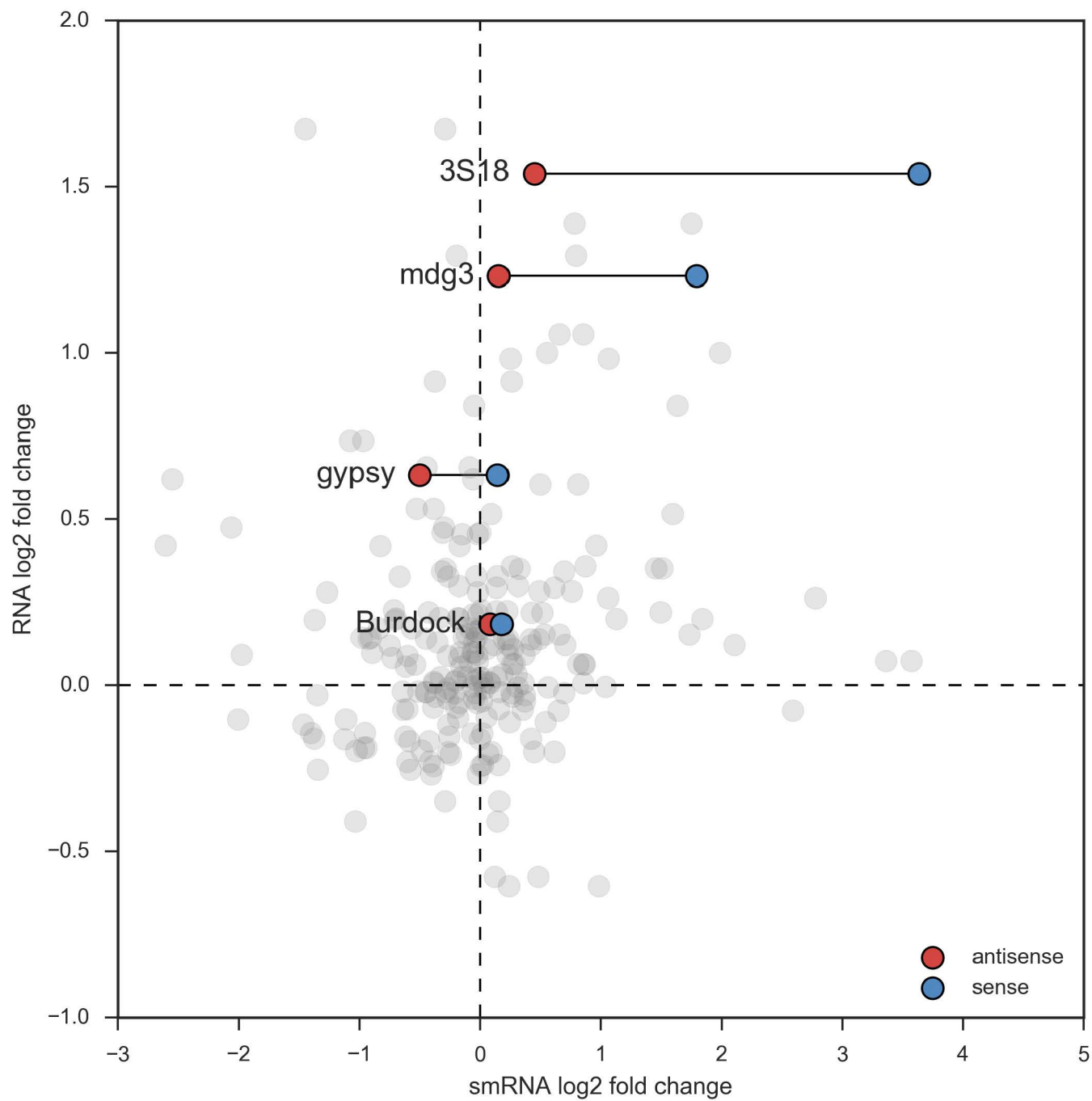

**b**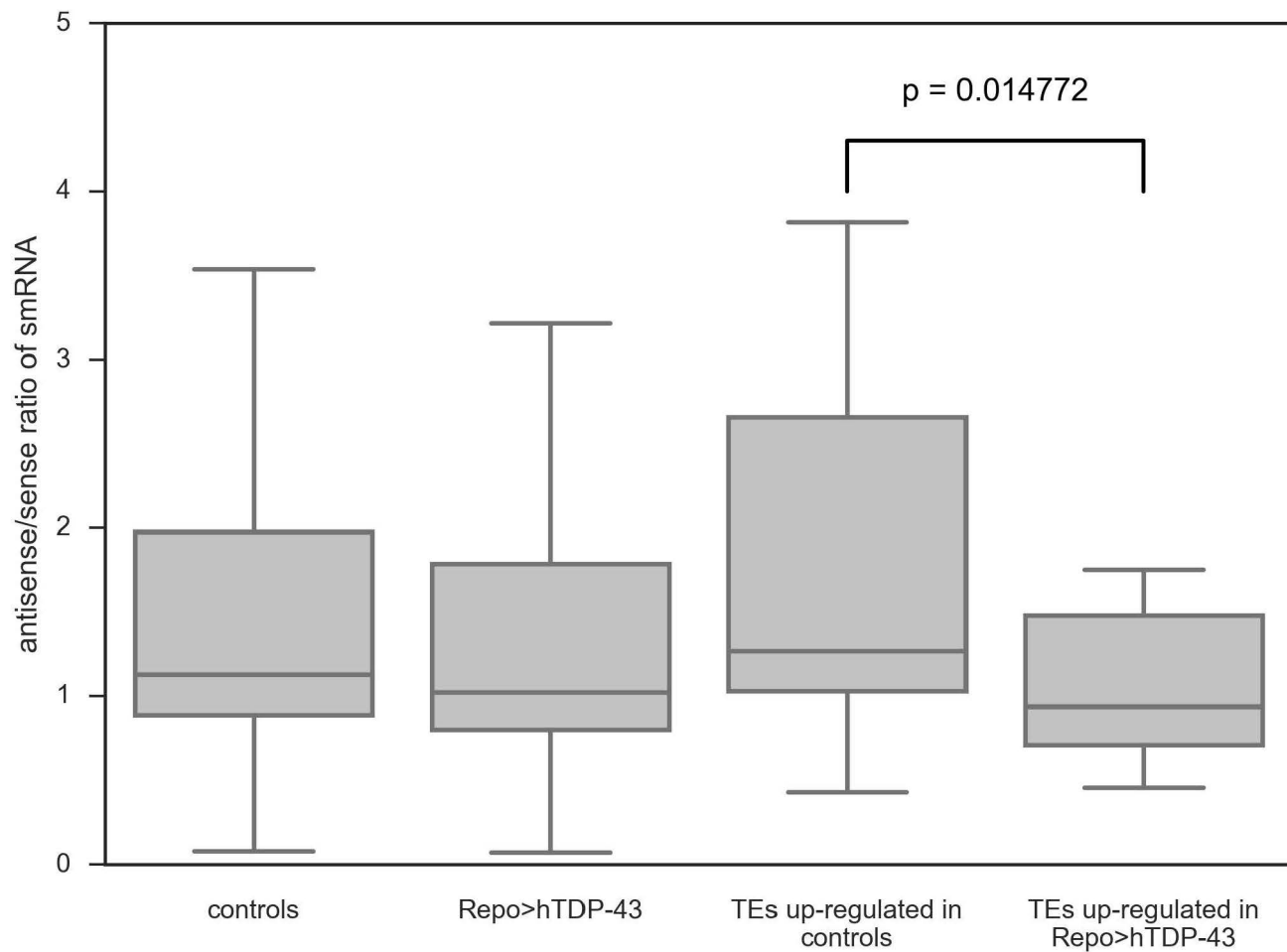

Supplement: S7 Fig — Sequencing of small-RNAs from heads detects siRNAs that are predicted to target broad range of RTEs. Among RTEs whose expression is elevated in Repo>TDP-43 (vs control), there is a selective decrease in anti-sense relative to sense stranded siRNAs. (A) scatter plots of log2Fold change in RNAseq vs log2Fold change in small-RNA seq. 3S18, mdg3 and gypsy exhibit elevated levels of expression in the RNAseq data (see Fig 1), and also exhibit decreased anti-sense/sense siRNA ratio. This is in contrast to Burdock, whose RNA levels are unchanged and whose siRNA anti-sense/sense ratio is unchanged. (B) Overall, there is a significant decrease in anti-sense/sense ratio for the subset of siRNAs that map to TEs whose levels are altered by Repo>hTDP-43. (PDF) [file pgen.1006635.s007.pdf]
