# Supplementary material for: Retrotransposon activation contributes to neurodegeneration in a Drosophila TDP-43 model of ALS
Source: PLoS Genet. 2017 Mar 16;13(3):e1006635. doi: 10.1371/journal.pgen.1006635 (PMC5354250; doi:10.1371/journal.pgen.1006635)
Supplement: S5 Fig — (A) An equivalent analysis as described for (S5A) demonstrates that neuronal (ELAV > loki(IR)) and glial (Repo > loki(IR)) expression of an IR directed against loki (loki(IR)) effectively blocks the age-dependent elevation of loki transcript levels, resulting in an ~2-fold reduction at 28 days post-eclosion. A one-way ANOVA shows a significant effect of genotype (p = 0.0039). N = 3–4 biological replicates. (B) Co-expression of GFP(IR) with hTDP-43 under Repo-Gal4 (Repo > hTDP-43 + GFP(IR)) does not significantly alter the number of TUNEL-positive nuclei detected compared to hTDP-43 expression alone under Repo-Gal4 (Repo > hTDP-43). N = 8 for Repo > hTDP-43 and N = 9 for Repo > hTDP-43 + GFP(IR); data normalized to Repo > hTDP-43. (C) qPCR for hTDP-43 expression (TARDBP) on whole head tissue demonstrates that co-expression of each of the IR constructs with hTDP-43 under Repo-Gal4 (Repo > hTDP-43 + GFP(IR), Repo > hTDP-43 + gypsy(IR), and Repo > hTDP-43 + loki(IR), respectively) does not significantly reduce hTDP-43 expression levels compared to hTDP-43 expression alone under Repo-Gal4 (Repo > hTDP-43) Fold change is displayed as the mean fold change relative to Repo > hTDP-43, while p-value represents the p-value of a two-tailed Student’s t-test in comparison to Repo > hTDP-43. N = 4 for all groups. (PDF) [file pgen.1006635.s005.pdf]

a.

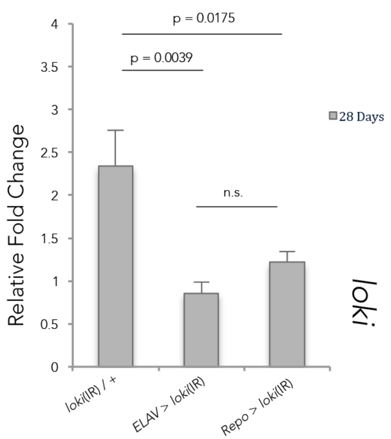

b.

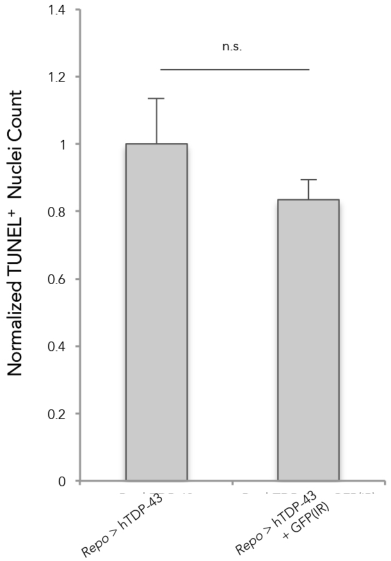

c.

TARDBP

Genotype	Fold Change	Variance	*p*-value
Repo > hTDP-43	1.000	± 0.093	1.000
Repo > hTDP-43 + GFP(IR)	1.649	± 0.042	0.017
Repo > hTDP-43 + *gypsy*(IR)	1.358	± 0.082	0.138
Repo > hTDP-43 + *loki*(IR)	0.702	± 0.041	0.165
